# Glassy cell carcinoma of the uterine cervix: a rare entity and literature review

**DOI:** 10.3332/ecancer.2025.1842

**Published:** 2025-02-06

**Authors:** Tenazoa-Villalobos José Richard, Yan-Quiroz Edgar Fermín, Ordoñez-Chinguel Augusto, Prado-Cucho Sofia Leonor, Miranda-Narro Adesman Isac, Vladimir Villoslada-Terrones

**Affiliations:** 1 Oncologic Surgery Area of the Víctor Lazarte Echegaray Hospital – EsSalud, Trujillo13013, Peru; 2 School of Medicine, Universidad Privada Antenor Orrego, Trujillo 13008, Peru; 3 Oncologic Surgery Area of the Virgen de la Puerta High Complexity Hospital – EsSalud, La Esperanza 13013, Peru; 4 School of Medicine, Universidad César Vallejo, Trujillo 13008, Peru; 5 Instituto Nacional de Enfermedades Neoplásicas – INEN, Lima 15036, Perú; 6 Faculty of Medicine, Universidad Peruana Cayetano Heredia, Lima 150135, Peru; 7 Faculty of Medicine, National University of Trujillo, Trujillo 13008, Peru; a https://orcid.org/0000-0003-3622-9408; b https://orcid.org/0000-0002-9128-4760; c https://orcid.org/0000-0001-8682-4948; d https://orcid.org/0000-0003-3042-0340; e https://orcid.org/0009-0003-2834-8092; f https://orcid.org/0000-0001-6877-5441

**Keywords:** glassy cell carcinoma, cervical cancer, chemotherapy, radiotherapy, radical hysterectomy

## Abstract

Glassy cell carcinoma is an extremely rare entity that occurs in 1% to 2% of all cases of cervical cancer, affects young women with greater predisposition, is related to poor prognosis, and distant metastasis. It correlates strongly with the presence of high-risk human papillomavirus (serotypes 16, 18 and 31) and histologically manifests as ground-glass cells, cytosol with vast granular and dense chromatin with large nuclei and protruding nucleoli. We present a 51-year-old woman who was diagnosed with glassy cell carcinoma of the cervix (before the latest edition of the World Health Organisation classification of tumours) in FIGO stage IB1 that was managed with radical hysterectomy and bilateral pelvic lymph node dissection, whose pathological result shows infiltration of the upper third of the vagina, changing the staging to FIGO IIA-1. She received adjuvant concurrent radiotherapy/chemotherapy with a good response, subsequent controls without signs of recurrence and remained currently alive.

## Introduction

Carcinoma of the uterine cervix ranks fourth among neoplasms affecting women and is the third leading cause of mortality among all gynecologic malignancies. During 2022, more than 660 thousand new cases were detected and more than 340 thousand deaths occurred globally, 85% of all these cases came from regions with limited resources. In developed countries, morbidity and mortality rates are low with respect to ovarian and endometrial cancer; however, in low-resource countries where screening and prevention programs are very poor; however, cervical cancer rates are high (it is the second most common cancer and the third leading cause of mortality), and human papillomavirus is an important cause for the development of this neoplasm and is detected in 99.7% of cases. The most common histological stripe found is squamous cell (70%) followed by adenocarcinoma (25%) [1–3].

Other histologic subtypes that comprise 3% to 5% of these cervical neoplasms are neuroendocrine, small cell, adenosquamous and glassy cell carcinoma. Glassy cell carcinoma is a very rare subtype, it is considered a variant of the adenosquamous type that represents less than 2%, is little reported and is histologically aggressive due to its rapid proliferation with a high risk of recurrence and metastasis. This neoplasm is known to affect young women an average of 10 years earlier than the other histologic strains and is closely related to the presence of HPV serotypes 16 and 18. Histopathologic analysis reveals an adenosquamous carcinoma with low histologic grade, scarce squamous cell differentiation and high mitotic rate. The glassy cells are characterised by large cells with ample cytoplasm, fine granules and ground glass appearance, enlarged nuclei with prominent nucleoli and thickened cell membrane. This glassy peculiarity manifests the vast chromatin of these cells [4–8].

To date, there are no studies that define standard management; however, the management applied is radical hysterectomy with bilateral pelvic lymph node dissection for early clinical stages of squamous cell carcinoma of the cervix based on the recommendations for the management of squamous cell carcinoma of the cérvix. However, because glassy cell carcinoma is rarely encountered, prospective and retrospective studies are scarce regarding management tactics; therefore, treatment in these cases is based on the same as for squamous cell carcinoma of the cervix [9].

## Case presentation

A 51-year-old woman from Huánuco, with no significant pathological history, gynecological history: Gravidity 5, Parity 5, first sexual intercourse at age 20, number of sexual partners 1, low socioeconomic level, with 1 year of illness, who manifests vaginal discharge, ‘like water from washing meat’, foul-smelling with clots, intense pelvic lancinating pain radiating to the lumbar area associated with dyspareunia and weight loss of approximately 10 kilos. She was seen at the health center where she was evaluated by a physician, who after finding an exophytic and bleeding tumour in the uterine cervix of 3 cm, decided to take a biopsy on 11 August 2015, the result of which showed Infiltrating Epidermoid Carcinoma.

**Figure 1.**
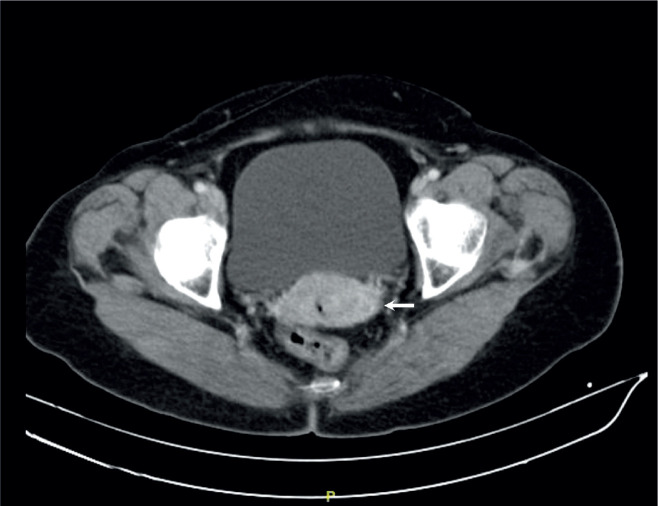
Abdominal and pelvic tomography with contrast, coronal section. There is contrast-enhancing tumour formation predominantly in the lower lip of the cervix of 4 cm (arrow), there is no involvement of the parametria or adenopathies suggestive of secondary involvement.

With such results, she was referred to the National Institute of Neoplastic Diseases – Lima, Peru and in August 2015 was admitted to the Department of Gynecologic Oncology, being approached integrally and the physical examination found normal external genitalia, wide elastic vagina without lesions, in the cervix was observed exophytic tumour, bleeding, stony, mobile, irregular predominantly in the posterior lip with proliferative lesion extending to the posterior cul-de-sac measuring 3.8 × 3 cm. Digitorectal examination: Parametrium not compromised by neoplasia. It is complemented with imaging studies: Tomography 22.08.2015: Tumour-like cervix measuring 4 cm in transverse diameter, there is associated alteration of the adjacent fatty planes. There is no evidence of retroperitoneal adenopathy in the iliac, mesenteric or inguinal chains. No signs of hydronephrosis. There is no evidence of free liquid in the abdomino-pelvic cavity. Bone structures without lytic or blastic lesions are evident. Soft tissues without alterations (Figure 1).

The patient was diagnosed with epidermoid carcinoma of the cervix FIGO IB2, and elective surgery was decided. She was admitted to the operating room on 25.09.2015 where exploratory laparotomy plus radical hysterectomy type C3 and bilateral pelvic lymphadenectomy were performed. In the pelvic examination under anesthesia, a 3.8 cm exoendophytic tumour of the cervix was palpated, friable and bleeding on contact with the left vaginal fornix. Right parametrium without evidence of involvement by the tumour and shortened left parametrium, not involved by the tumour. When the pelvic cavity is opened there is no evidence of free fluid, no carcinomatosis. Uterus 6 × 4 cm, not myomatous, smooth surface. Cervix occupied by neoplasia. Fallopian tubes and ovaries 2 × 1 cm, no signs of tumour infiltration. The thickened parametrium is observed, and no pelvic or para-aortic lymphadenopathies are palpated. After surgery, the patient remained in the hospital where she evolved favourably and was discharged on postoperative day 3.

**Figure 2.**
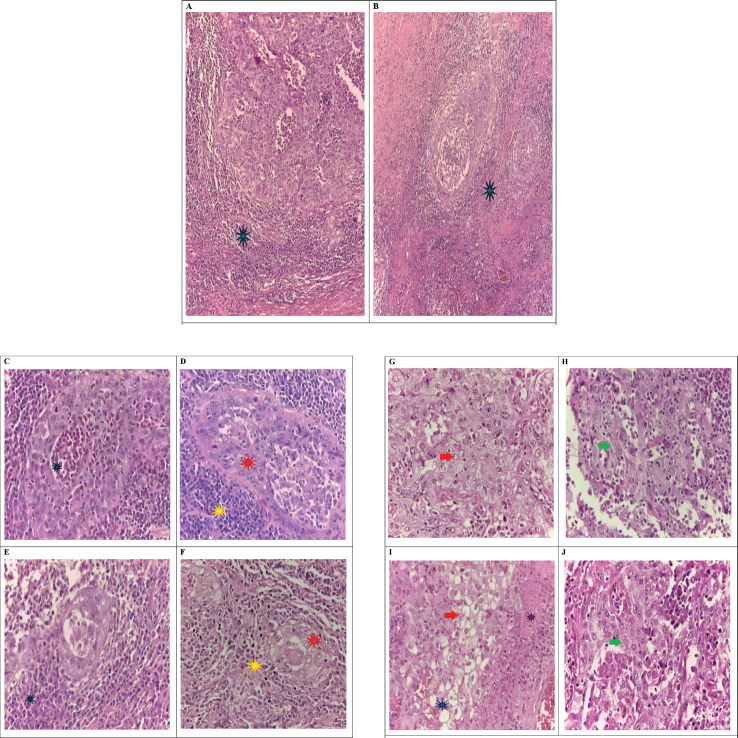
Microscopic image. Hematoxylin & Eosin, magnification 4X: In image A and B epithelial tumour cells arranged in sheets (A) or groups (B) and surrounding these groups there is a dense inflammatory infiltrate (*) and necrotic debris. Focal keratin production and areas of glandular differentiation can also be seen. Images C, D, E and F show tumour cells arranged in nests or glandular structures, which are surrounded by a dense inflammatory infiltrate formed by abundant eosinophils (*), lymphocytes, plasmacytes (*), neutrophils and necrotic debris. The cells present vesicular, round or oval nuclei, with one or more prominent nucleoli (*). In images G, H, I and J tumour cells are observed arranged in a solid pattern (à) or forming pseudoglands (à),these cells are surrounded by dense inflammatory infiltrate and necrotic debris(*). The chromatin of the nuclei varies from finely dispersed to coarse and irregular (G), the cells also show cytoplasmic vacuolisation and some cells may be multinucleated. Mitotic and apoptotic activity is frequent (H). Foci of individual keratinisation can be observed in areas (J) and focal differentiation of clear cells can also be seen (I*).

Pathological anatomy results were obtained: infiltrating epidermoid squamous cell carcinoma of the uterine cervix (Figure 2), tumour size of 3.7 × 3.2 × 1.7 cm histological grade poorly differentiated, stromal invasion of 17 mm, free surgical margin, absent vascular permeation, free uterine body, with atrophic endometrium, free parametrium, vagina involved in proximal third with remaining free surgical margins, free right and left adnexa, lymph node dissection: right pelvic lymph nodes and left pelvic lymph nodes free of neoplasia (0/33).

Taking into account the pathology results and the involvement at the level of the vagina is classified as a glassy cell epidermoid carcinoma of the cervix FIGO IIA-1, it was decided to perform multidisciplinary management together with the radiotherapy and medical oncology services, taking into account the histological type (glassy cells), which is a histological variant with a more aggressive clinical course; was scheduled for external radiotherapy in lateral pelvic fields at a dose of 5,000 cGy in 25 sessions, in concurrence with chemotherapy (Carboplatin 2 AUC: 246 mg weekly for 5 doses), and subsequent to concurrence patient received a booster with Brachytherapy in January 2016 continued chemotherapy until May 2016 with Carboplatin/Paclitaxel. During treatment, the patient remains hemodynamically stable with few adverse effects, with good tolerance to interdisciplinary management showing good response. Contrast tomography of the abdomen and pelvis was performed on 05.04.2016 where no retroperitoneal or mesentery lymphadenopathies were observed, absence of internal genitalia due to surgical history, showing vaginal vault with preserved density and morphology without evidence of pathological enhancement with contrast that suggests recurrence of the disease. No free liquid is observed in the cavity.

The patient attends controls strictly by the outpatient clinic with imaging controls and a Pap smear of the vaginal vault with negative results of dysplastic cells. The patient has lost her sight since October 2019, she states that she does not go to the office due to the COVID-19 pandemic. She returns for check-ups on 18.12.2023, reports being asymptomatic, denies vaginal bleeding and denies pelvic pain. Physical examination shows preserved external genitalia, median vagina little elastic and vaginal vault without lesions. Digitorectal examination showed no palpable tumours. Ultrasound of the abdomen and pelvis was performed on 9 April 2024 showing liver with preserved morphology, without focal lesions inside, kidneys and spleen without evidence of lesions, absence of internal genitalia and no free liquid in the cavity. Chest X-ray 10.04.2024 shows expanded lungs without lesions and preserved vascular structure. Pap smear negative for dysplastic cells. She was evaluated again on 16 September 2024, and no signs of recurrence of the disease were found, so she will be monitored annually due to the favourable evolution of the disease.

## Discussion

Glassy cell carcinoma is extremely rare, constituting between 1% and 2% of all cases of malignant cervical neoplasia, it does not usually occur in the presence of other malignant strains and compared to other cervical cancers, it tends to appear in younger patients between the third and fourth decade of life, which is 10 years younger than the average age of all women with malignant cervical neoplasia, and few cases have been reported in the last 10 years (Table 1). In most cases, patients present with advanced cervical neoplasia, which is associated with an ominous prognosis, is radioresistant and has high rates of metastasis [6, 10, 11]. Squamous cell carcinoma was first described in 1956 by Cherry and Glucksmann [8] where it is specifically described and distinguished from poorly differentiated adenosquamous types. Viewed microscopically these glassy cells are distinguished by abundant cellularity, tarnished glassy cytosol with fine granules, cell wall that stains with Eosin with distinct borders and prominent nuclei that have abundant irregular and coarse chromatin with prominent nucleoli, that many times can be confused with inclusions of the herpes virus or Reed Stenberg cells, in addition it can be noted that there is cytosolic vacuolisation, other cells with multiple nuclei in abnormal forms with many mitotic figures and a dense inflammatory infiltrate with eosinophils and plasmacytes [7, 8, 12–15].

In 1976, an exhaustive investigation of glassy cell carcinoma was carried out resulting in the reformulation and broadening of histological diagnostic guidelines. As a result, this malignant neoplasm has undergone changes in the last two World Health Organisation classifications defining it as ‘other epithelial tumours’ (in the fourth edition of 2014) and the most recent (fifth edition of 2020) discourages its use as ‘glassy cell carcinoma’ revoking it as a specific histologic subtype [7, 16, 17].

The presence of high-risk HPV serotypes 16, 18 and 32 are closely linked to vitreous cell carcinoma, as are adenocarcinoma and squamous cell carcinoma with HPV infection. Women report that the predominant symptom is vaginal bleeding that lasts 4 months, this is because this neoplasm arises at the junction of squamous cells with columnar cells or can grow even in the endocervix and extends into the upper third of the vagina or to the cardinal ligaments or Mackenrodt's ligaments. After the primary treatment, local recurrence usually occurs in the vaginal stump or in the retroperitoneal lymph nodes within 24 months, and it is worth mentioning that most of the distant metastases occur 31 months after the treatment, most frequently in the lung, liver, followed by the spleen and skeleton [7, 10, 18–20].

The management of glassy cell carcinoma is by radical hysterectomy together with pelvic lymph node dissection which is considered the treatment of choice, patients requiring adjuvant management are those with at least one of the following high-risk factors such as a large lesion, nodal involvement, cardinal ligament involvement and compromised surgical margin. They also include intermediate risk factors such as lymphovascular invasion, stromal invasion and tumour size of 3 cm. Adjuvant radiotherapy has been shown to improve overall survival and decrease disease-free survival, applying the same radiotherapy principles of cervical cancer management for glassy cell carcinoma, in concurrence with chemotherapy [21–26].

Glassy cell carcinoma has a bleak prognosis; however, this bleak outlook has improved thanks to multimodal therapy and overall survival at 5 years is currently 80% for FIGO clinical stages I and II, the results of which are encouraging and considered curable [9, 19].

## Conclusion

Glassy cell carcinoma is a subject that needs to be studied extensively; thanks to multimodal management, it is possible to ensure optimal survival in patients who are diagnosed, as well as to reinforce screening strategies and early diagnosis. In the case presented, the patient has a favourable evolution with a survival that exceeded 5 years thanks to the integrated management that was put in place. Unfortunately, there are not many studies on glassy cell carcinoma, so retrospective analyses are necessary.

## Conflicts of interest

There are no conflicts of interest for the authors.

## Funding

The authors are self-financing this study.

## Ethical approval

The patient signed and approved the written informed consent for the publication of the presented case report, as well as the attached images. The Ethics Committee of the National Institute of Neoplastic Diseases approved the publication of this case.

## Author contributions

JRTV Y EFYQ Preparation, creation and presentation of the paper, drafting of the manuscript and supervision. AOC & SPC Provision of anatomic pathology materials and drafting of manuscript. VVT and AIMN drafting of manuscript. JRTV & EFYQ Approval of manuscript submission.

## Figures and Tables

**Table 1.** Clinical details, management and survival of cases reported in Pubmed of glassy cell carcinoma in the last 10 years.

Author	Year	Age	FIGO Stadium	Treatment	Survival
Habara *et al* [27]	2023	60	IB1	Surgery: Radical hysterectomy Chemotherapy: No Radiotherapy: No	Patient alive 31 months after treatment
Tasaki *et al* [28]	2021	30	IIB	Surgery: Radical hysterectomy Chemotherapy: Yes Radiotherapy: Yes	Recurrence 9 months after adjuvant treatment, pulmonary metastasis and carcinomatous meningitis, died 1 week later.
Kazakova *et al* [29]	2020	67	IA	Surgery: Radical hysterectomy Chemotherapy: Yes Radiotherapy: Yes	Patient alive 18 months after treatment
Yoon *et al* [30]	2016	38	IIB	Surgery: No Chemotherapy: Yes Radiotherapy: Yes	Patient alive at 9 years after treatment
		63	IIB	Surgery: No Chemotherapy: Yes Radiotherapy: Yes	Patient alive at 8 years after treatment
		36	IIB	Surgery: Sigmoid colostomy due to rectovaginal fistula after radiotherapy. Chemotherapy: Yes Radiotherapy: Yes	Patient with recurrence of large pelvic mass at 9 years after treatment, bilateral pelvic and inguinal adenopathies. Died 1 year after tumor recurrence.
		67	IIIB	Surgery: No Chemotherapy: Yes Radiotherapy: Yes	Patient presented gastrointestinal bleeding during concurrent chemoradiotherapy, died of hypovolemic shock.
		37	IIB	Surgery: No Chemotherapy: Yes Radiotherapy: Yes	Patient alive 2 years after treatment
McEachron *et al* [31]	2016	16	IVB	Surgery: No Chemotherapy: Yes Radiotherapy: Yes Immunotherapy: Yes, cetuximab and erlotinib.	Patient did not complete therapy due to side effects. He died shortly after discontinuation of therapy.
Carocha *et al* [32]	2015	34	IB1	Surgery: Radical hysterectomy Chemotherapy: Yes Radiotherapy: Yes	Patient presented perineal recurrence in vulva and right inguinal adenopathies. He died 9 months after treatment.
